# Explainable AI for suicide risk detection: gender- and age-specific patterns from real-time crisis chats

**DOI:** 10.3389/fmed.2025.1703755

**Published:** 2025-12-18

**Authors:** Meytal Grimland, Moran Liberman, Hadas Yeshayahu, Joy Benatov, Noam Munz, Avi Segal, Loona Ben Dayan, Inbar Shenfeld, Kobi Gal, Yossi Levi-Belz

**Affiliations:** 1 The Lior Tsfaty Center for Suicide and Mental Pain Studies, University of Haifa, Haifa, Israel; 2 Department of Behavioral Sciences, Ruppin Academic Center, Emek Hefer, Israel; 3 Department of Special Education, University of Haifa, Haifa, Israel; 4 Department of Software and Information Systems Engineering, Ben-Gurion University of the Negev, Beer-Sheva, Israel; 5 SAHAR, Online Mental Support, Tel Aviv, Israel; 6 School of Informatics, University of Edinburgh, Edinburgh, United Kingdom; 7 Faculty of Education, Department of Counseling and Human Development, University of Haifa, Haifa, Israel

**Keywords:** explainable AI, natural language processing, suicide prevention, crisis helpline, gender and age differences

## Abstract

**Background:**

Suicide remains a leading cause of death worldwide, yet conventional risk models based on static demographic or diagnostic factors show limited predictive value. Advances in explainable artificial intelligence (AI) and natural language processing (NLP) offer new opportunities for real-time, personalized risk detection.

**Methods:**

We analyzed 17,564 chat sessions (2017–2021) from Sahar, a digital crisis helpline. Suicide risk (SR) was defined by explicit suicidal ideation. A theory-driven lexicon of 20 psychological constructs (e.g., hopelessness, loneliness, self-harm), derived from leading SR frameworks, was applied using NLP. Logistic regression models estimated associations between constructs and SR, stratified by gender and age (10–17, 18–20, 21–40, and 41+). Temporal trajectories of predictors were examined across five conversation stages.

**Results:**

Previous suicide attempts and hopelessness were the strongest predictors across all groups. Gender differences emerged: among women, loneliness was a consistent predictor, whereas in men, thwarted belongingness and late-session depression were more salient. Age analyses showed developmental specificity: prior attempts were strongest in adolescents, hopelessness and self-harm peaked in young adults, thwarted belongingness strengthened with age, and loneliness predicted risk only among adults aged 41+. Several factors, including bullying/cyberbullying, LGBTQ identity, and perfectionism, were inversely associated with SR in specific subgroups.

**Conclusions:**

This study demonstrates how explainable, theory-informed NLP can capture dynamic SR factors in real-world crisis interactions. Findings reveal distinct gender- and age-specific pathways, underscoring the need for personalized prevention strategies. Beyond theoretical insights, the approach highlights the potential of AI-driven, interpretable monitoring tools to support crisis counselors in detecting escalating risk earlier and tailoring interventions. Such methods can enhance the accuracy, timeliness, and equity of suicide prevention, and illustrate how explainable AI can translate psychological theory into clinically meaningful tools for mental health screening and early intervention.

## 1 Introduction

Suicide remains one of the most pressing global public health challenges, with more than 700,000 lives lost annually (1). These figures highlight not only the urgency of prevention but also the need to improve how suicide risk (SR) is identified and managed in real time. Traditional prediction models, often grounded in static demographic or diagnostic factors, have shown limited accuracy and clinical utility (2, 3). SR is inherently dynamic, fluctuating rapidly in response to acute psychological states and situational stressors. To address this gap, there is growing recognition that innovative, technology-supported approaches are needed to move beyond static predictors toward tools that can provide timely, individualized insights into risk.

Advances in explainable artificial intelligence (AI) and natural language processing (NLP) offer new opportunities for screening and early detection of SR in healthcare and crisis settings (4, 5). Unlike black-box models, explainable AI methods can integrate established psychological theories with transparent algorithms, enabling both accuracy and interpretability (6). Such approaches can bridge the gap between data-driven advances and clinical practice by allowing clinicians and primary care providers to understand why a model signals elevated risk and to act accordingly (7). Importantly, demographic variation—particularly in gender and age—shapes how SR is experienced and expressed, underscoring the need for explainable, personalized tools that can support context-sensitive assessment and intervention.

Gender differences are well-documented in what has been termed the “gender paradox”: men are more likely to die by suicide, while women are more likely to attempt it (8, 9). This disparity has prompted growing recognition that, in addition to general risk factors such as psychiatric illness, self-harm history, and recent life stressors (3), gender-specific mechanisms also influence suicide vulnerability. For instance, men are more prone to externalizing symptoms such as aggression and impulsivity, often leading to more lethal methods (10), whereas women typically use less lethal methods, contributing to lower suicide mortality (11). Furthermore, the psychological expression of risk factors such as hopelessness or social disconnection may differ by gender. While both men and women may experience these states, their communication, intensity, and behavioral outcomes vary (12). Thus, gender-sensitive assessment frameworks are necessary to capture these nuanced differences and enhance prevention strategies.

Age is another critical dimension in SR, with distinct developmental stages linked to different vulnerabilities (13). Among adolescents (10–17), specific risks include peer dynamics, academic stress, identity formation, and increased exposure to online harassment and cyberbullying (14, 15). Young adults (18–20), in contrast, navigate major life transitions—educational, occupational, and relational—which can heighten susceptibility to feelings of thwarted belongingness and perceived burdensomeness (16). Adults aged 21–40 face multifaceted pressures from career demands, financial stress, and family responsibilities, which interact in complex ways to influence mental health (17). In older adults (41+), SR is more closely tied to chronic isolation, hopelessness, and deteriorating health, often exacerbated by the loss of social roles or diminishing support networks (18, 19). These patterns call for age-informed assessments that account for evolving psychological and situational stressors throughout the lifespan.

Crisis hotlines play a critical role in suicide prevention by offering immediate support and connecting individuals in distress to mental health services (20, 21). In recent years, chat-based formats have gained prominence due to their accessibility, anonymity, and reduced stigma, making them particularly appealing to those reluctant to seek traditional help (22–24). They provide a unique window into the real-time manifestation of SR factors. These interactions generate substantial data that, when analyzed systematically, may reveal important patterns in how psychological distress is expressed across different demographic groups (23). The development of robust analytical frameworks for examining these communications can enhance our ability to identify and respond to SR effectively. Moreover, the complexity of SR assessment, particularly in the context of crisis communications, necessitates innovative methodological approaches that can capture both psychological and demographic patterns of risk. The integration of computational methods with psychological theory offers promising opportunities for advancing our understanding of how risk factors manifest across different populations and contexts (25).

To operationalize these theoretical insights in language form, the lexicon used in this study was informed by leading psychological and clinical frameworks of SR. The Interpersonal Theory of Suicide (26, 27) provides constructs such as perceived burdensomeness and thwarted belongingness, which capture key interpersonal and cognitive–affective drivers of suicidal desire. The Suicide Crisis Syndrome (28) describes an acute pre-suicidal state marked by affective disturbance, loss of cognitive control, hyperarousal, and social withdrawal, offering a framework for identifying crisis-related emotional and cognitive dysregulation in language that may emerge during suicidal crises. Complementing these models, the Columbia Suicide Severity Rating Scale (C-SSRS) offers validated descriptors of suicidal thoughts, intent, and planning, which guided the representation of explicit suicidal ideation and related expressions (29). Together, these frameworks informed the development of theory-based linguistic categories that capture both chronic vulnerabilities and crisis markers, ensuring that the lexicon reflects well-established psychological and clinical dimensions of SR as they manifest in real-time chat communication.

### 1.1 The present study

Building on our prior work that identified overall psychological predictors of SR in real-time chats (30), the present study extends this framework by examining how SR manifests across gender and age groups. Integrating psychological theory with explainable AI methods we draw on a large dataset of anonymized digital crisis chats to explore demographic differences in the language of SR. We applied a transparent natural language processing (NLP) pipeline built on a theory-driven lexicon of psychological constructs (e.g., hopelessness, loneliness, self-harm). This approach aligns with recent developments in explainable AI for mental health, which emphasize transparency and interpretability as prerequisites for trustworthy clinical applications (31). Unlike opaque machine learning models, this lexicon-based, logistic regression framework allows the risk contributions of each construct to be quantified and directly interpreted by clinicians and researchers. In this way, the approach functions as a form of explainable AI that translates established psychological theories into interpretable digital markers of SR.

Our analyses pursued two key goals. First, we assessed whether specific psychological constructs were differentially predictive of SR across gender and developmental groups (10–17, 18–20, 21–40, and 41+). Second, we examined temporal trajectories of these predictors across the course of conversations to capture how risk unfolds dynamically in real-world interactions. By bridging theory-driven frameworks with interpretable, data-driven methods, this study demonstrates how explainable NLP can provide clinically meaningful insights into SR while preserving transparency for practitioners.

Ultimately, the findings are intended to support the development of AI-assisted screening and monitoring tools that can be integrated into digital crisis services and, more broadly, into healthcare workflows. By offering interpretable, context-sensitive indicators of SR, such tools may enhance early detection, empower providers with actionable insights, and improve the timeliness and accuracy of suicide prevention strategies.

## 2 Materials and methods

### 2.1 Sample

This study analyzed 17,564 chat sessions from Sahar, a human-staffed, real-time online emotional-support and crisis helpline that provides anonymous, text-based support in Hebrew and Arabic (only Hebrew chats were included). The helpline is staffed by trained volunteers and each duty shift is supervised by a licensed mental health professional who provides consultation and guidance in complex or high-risk chats. This supervision structure supports early detection of escalating distress and enables timely intervention when SR is suspected. Sessions were conducted between 2017 and 2021, each lasting up to 40 min. As part of routine practice, volunteers document each conversation and assign broad presenting-issue labels (e.g., anxiety, depression, loneliness, interpersonal conflict, suicidal ideation) that summarize the main concerns raised during the chat for internal monitoring purposes. In this study, these service-level labels were used solely to identify chats containing explicit suicidal ideation and were not included as predictors in the lexicon-based analyses. Volunteers receive structured training and ongoing supervision from licensed mental-health professionals to promote consistency in documenting presenting issues. SR was defined as any session in which the user expressed suicidal ideation (to be defined in the outcome section). Of the total sample, 17% (*n* = 3,097) were classified as SR. To assess the reliability of this classification, 600 sessions (200 non-suicidal and 400 suicidal) were randomly selected and independently reviewed in a blinded process by three clinical psychologists specializing in suicide prevention. Inter-rater agreement yielded a Cohen's kappa of 0.731, indicating substantial reliability.

Demographic subgrouping was based on user-reported gender (binarily defined) and age, categorized into four developmental groups: 10–17 (school age), 18–20 (mandatory military service), 21–40 (young and middle adulthood), and 41+ (older adulthood).

### 2.2 Outcome measure

The primary outcome was SR, defined as the presence of explicit expressions of suicidal ideation within the chat session. In Sahar's routine documentation procedures, trained volunteers label a chat as SR when the user directly communicates thoughts of ending their life. Examples of statements that meet this criterion include: “I feel like I want to end my life,”, “I keep having thoughts that it would be better if I died,”, “I'm thinking about killing myself.” This operational definition captures clear, direct expressions of suicidal ideation and distinguishes SR from general emotional distress.

### 2.3 Explanatory variables

Suicide risk Factors-Based Lexicon (SRF): the primary predictor variables were language representations of psychological risk factors, encoded by a suicide risk factor (SRF) lexicon. The lexicon included both theory-based psychological constructs and empirically supported psychosocial risk factors. The theory-based constructs captured core mechanisms derived from leading models such as the Interpersonal Theory of Suicide, for example, perceived burdensomeness and thwarted belongingness. Complementing these were empirically validated psychosocial and behavioral indicators documented in prior research, including prior suicide attempt, deliberate self-harm, depressive symptoms, psychopathology, family suicide history, exposure to bullying, sexual harassment, adverse life events, LGBT-related and immigration-related stressors, impulsivity, and perfectionism. This combined framework allows the model to capture both explanatory and applied dimensions, showing how psychological processes and behavioral markers jointly contribute to SR in real-time conversations.

Lexicon development followed three steps. First, we generated language representations for the psychological constructs and empirically supported risk indicators described above. Second, we drew additional expressions from validated questionnaires tapping these theories and constructs [e.g., the interpersonal needs questionnaire (INQ-15)], adapting items to conversational phrasing where appropriate. Third, we reviewed 200 randomly selected suicide-risk chats to identify naturally occurring linguistic variants and refine phrasing to the helpline context. The resulting lexicon contains 20 conceptually distinct categories, with phrase sets ranging from 20 to over 400 items per category. For a detailed description of the lexicon development process and its theoretical foundations, see Grimland et al. (30).

### 2.4 Language processing and lexicon implementation

Chat transcripts were cleaned before analysis to remove non-textual elements and standardize formatting. No stemming, lemmatization, or fuzzy matching was applied, as the lexicon was designed to capture the natural phrasing and linguistic variants typical of real-time chat language. Lexicon phrases were implemented using regular expressions that accounted for common variations in wording and punctuation while maintaining precise matching. For each chat, we calculated the frequency of expressions corresponding to each lexicon category, resulting in a vector representation in which each element reflects the relative presence of a given risk factor. These vectors served as model input features for subsequent logistic-regression analyses. We then trained a logistic regression model that uses category frequencies as features.

### 2.5 Statistical analysis

**Figure 1:**
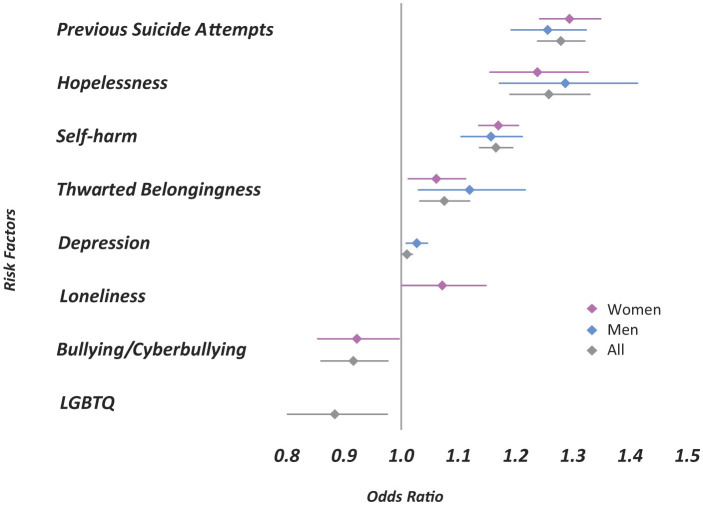
Statistically significant predictors of SR by gender (*p* < 0.05). Pink diamonds represent women, blue diamonds represent men, and gray diamonds represent the overall sample. Horizontal lines indicate 95% confidence intervals. The vertical reference line at 1.0 represents no association; values greater than 1.0 indicate increased odds of suicide risk, while values less than 1.0 indicate protective effects.

We performed logistic regression analyses to assess the association between psychological constructs and SR. Odds ratios (ORs) with 95% confidence intervals (CIs) quantified the strength of these associations. Because the models rely on theory-driven predictors whose coefficients can be directly interpreted, this approach functions as an explainable AI method that emphasizes transparency and interpretability over black-box prediction (31, 32). To examine gender-specific patterns, we estimated one model for the full sample (“All”) and two gender-stratified models (“Women” and “Men”). Figure 1 presents the significant predictors from each model. These reflect within-group associations; no formal tests comparing coefficients across genders were conducted. In addition, analyses were stratified separately by gender and by age group. SR base rates for every subgroup are reported in Table 1 to allow inspection of sample size and potential imbalance within each model. We also conducted temporal analyses examined changes in the predictive power of psychological factors across chat intervals (20%, 40%, 60%, 80%, and 100%) to capture how risk-related language evolves during conversations and to identify factors that may signal elevated risk early in the exchange. All models included an intercept (constant term). Model calibration was assessed using the Hosmer–Lemeshow goodness-of-fit test (χ^2^(8) = 11.21, *p* = 0.19), indicating adequate fit. All analyses were conducted using the Python library statsmodels.

**Table 1:** Sample size and suicide risk prevalence by gender and age group.

**Subgroup**	**Total chats (*N*)**	**SR cases (*n*)**	**SR prevalence (%)**
All chats	17,564	3,097	17.5
**By gender**			
Female	12,074	2,173	18.0
Male	5,343	908	17.0
Unknown/not stated^*^	147	16	11.0
**By age group**			
10–17	4,179	627	15.0
18–20	3,283	671	20.5
21–40	8,603	1,482	17.2
41 +	1,467	314	21.4
Unknown/not stated^*^	32	3	9.3

^*^Unknown/not stated cases excluded from gender- and age-specific analyses.

## 3 Results

### 3.1 Gender differences in suicide risk factors

To assess the predictive value of each factor representing a theory-driven psychological construct, we conducted two logistic regression analyses with SR as the dependent variable (Figure 1). To complement the odds-ratio interpretation, we also evaluated model performance on a held-out test set. The model demonstrated good discriminative ability (ROC–AUC = 0.847), with precision of 72.8%, recall of 48.4%, F1 of 58.1, and F2 of 51.9. The results revealed distinct patterns of association by gender. Among women, the strongest predictor of SR in chats was a history of previous suicide attempts (OR = 1.29, 95% CI = 1.24–1.35), followed by hopelessness (OR = 1.24, 95% CI = 1.16–1.33) and self-harm (OR = 1.17, 95% CI = 1.14–1.20). Then loneliness (OR = 1.07, 95% CI = 1.00–1.15) and thwarted belongingness (OR = 1.06, 95% CI = 1.01–1.11) which showed weak but significant associations with SR. In comparison, among men a slightly different hierarchy in the relevant factors that contributed to SR was noted, with hopelessness being the strongest predictor (OR = 1.29, 95% CI = 1.17–1.41), followed by previous suicide attempt (OR = 1.26, 95% CI = 1.19–1.32) and self-harm (OR = 1.16, 95% CI = 1.10–1.21). Thwarted belongingness also showed an association with SR chats in men (OR = 1.12, 95% CI = 1.03–1.22).

Two factors had different contributions for SR prediction. Depression was marginally predictive for men (OR = 1.03, 95% CI = 1.01–1.05) but non-significant for women. Loneliness showed a positive association with SR chats in women (OR = 1.07, 95% CI = 1.00–1.15) but was not significantly associated in men. Another factor that marked a difference between men and women was bullying/cyberbullying which was negatively associated with SR chats for women, indicating a possible predictor of emotional distress rather than SR (OR = 0.91, 95% CI = 0.85–0.97). Identifying as LGBTQ also exhibited significant odds ratios below 1.0 in the overall sample, implying greater uncertainty in its predictive role within female and male participants. All the other categories were insignificant and henceforth not mentioned.

### 3.2 Temporal trajectories of suicide risk factors by gender

**Figure 2:**
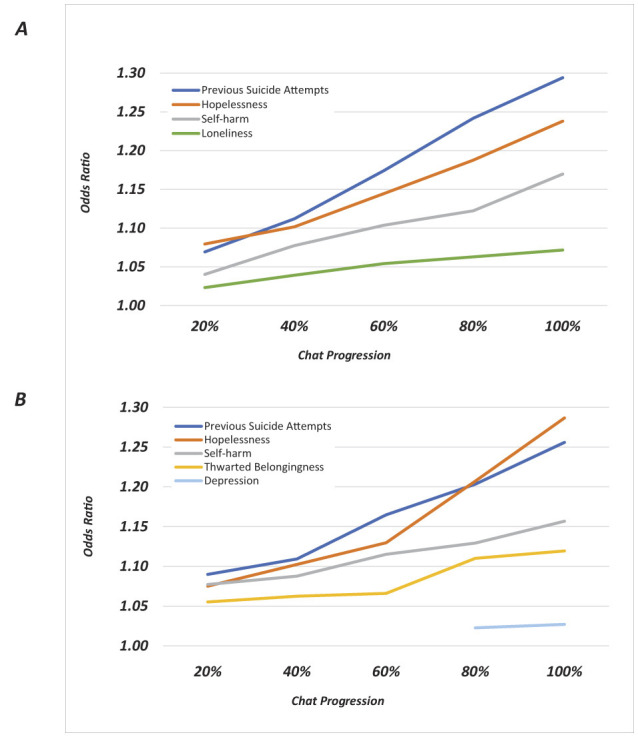
Temporal trajectories of statistically significant suicide risk predictors across chat progression (*p* < 0.05). **(A)** Women. **(B)** Men. Lines represent different psychological factors tracked at 20%, 40%, 60%, 80%, and 100% of chat completion. Trajectories demonstrate how associations between factors and suicide risk evolve throughout the conversation.

To explore the temporal progression of risk factors throughout the chat sessions, we examined how each factor's odds ratio changed at different stages of the conversation (20%, 40%, 60%, 80%, and 100%), stratified by gender (Figure 2A for women and Figure 2B for men).

The findings indicate that most factors followed consistent trajectories across the conversation timeline, with rising odds ratios as the session progressed. Notably, however, there were gender-specific differences. Among women, loneliness emerged as a modest but consistent predictor of SR throughout the conversation (20% OR = 1.02, 95% CI = 1.00–1.04; 40% OR = 1.04, 95% CI = 1.01–1.07; 60% OR = 1.05, 95% CI = 1.01–1.10; 80% OR = 1.06, 95% CI = 1.01–1.12; 100% OR = 1.07, 95% CI = 1.00–1.15). Among men, loneliness was not a significant predictor; instead, thwarted belongingness was significantly associated with suicidal ideation, with odds ratios increasing toward the later stages of the conversation (20% OR = 1.06, 95% CI = 1.02–1.09; 40% OR = 1.06, 95% CI = 1.02–1.11; 60% OR = 1.07, 95% CI = 1.01–1.12; 80% OR = 1.11, 95% CI = 1.04–1.19; 100% OR = 1.12, 95% CI = 1.03–1.22). Depression was significant only for men and only at the final stages of the conversation (80% OR = 1.02, 95% CI = 1.01–1.04; 100% OR = 1.03, 95% CI = 1.01–1.05).

### 3.3 Age differences in suicide risk factors

**Figure 3:**
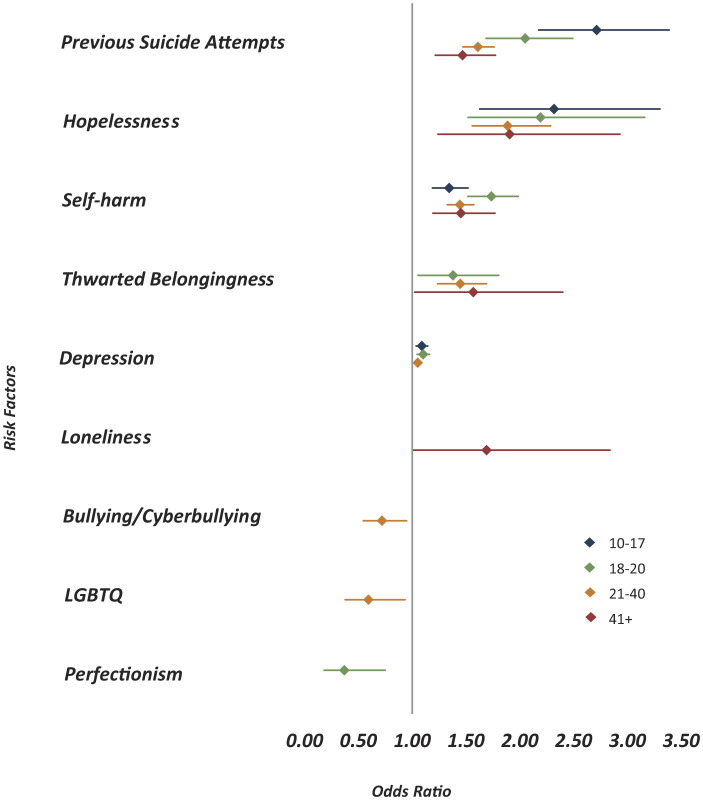
Statistically significant predictors of suicide risk across age groups (*p* < 0.05). Each color represents a different age group (10–17, 18–20, 21–40, and 41+). Horizontal lines indicate 95% confidence intervals. The vertical reference line at 1.0 represents no association; values greater than 1.0 indicate increased odds of suicide risk, while values less than 1.0 indicate protective effects.

Figure 3 presents odds ratios of suicide predictors stratified by age groups (10–17, 18–20, 21–40, and 41+), revealing distinct age-specific patterns. Previous suicide attempts consistently showed the strongest association with SR across all age groups, particularly among adolescents aged 10–17 (OR = 2.71, 95% CI = 2.17–3.39), with diminishing strength in older groups. Hopelessness was also a robust predictor across all ages, peaking in the 18–20 group (OR = 2.19, 95% CI = 1.52–3.16), and self-harm was also significant in all groups, with the highest odds observed in young adults aged 18–20 (OR = 1.74, 95% CI = 1.52–1.98). Thwarted belongingness emerged as a significant predictor from ages 18 and above, increasing in effect size with age. Interestingly, loneliness was only significant in the 41+ age group (OR = 1.69, 95% CI = 1.01–2.84), suggesting its heightened role later in life. In contrast, bullying/cyberbullying (OR = 0.72, 95% CI = 0.55–0.95) and LGBTQ identity (OR = 0.59, 95% CI = 0.38–0.93) were negatively associated with SR in the 21–40 age group, indicating that these factors may reflect emotional distress rather than SR in this demographic. Similarly, perfectionism was negatively associated with SR in the 18–20 group (OR = 0.37, 95% CI = 0.18–0.75), further emphasizing that not all distress markers carry the same risk implications across ages.

## 4 Discussion

This study demonstrates the value of explainable AI approaches for suicide prevention by integrating psychological theory with real-time crisis chat data. Through a transparent, lexicon-based NLP framework combined with logistic regression, we identified both shared and divergent predictors of SR across gender and age groups. Unlike black-box machine learning models, this approach provides interpretable results that can be readily understood by clinicians and counselors, highlighting not only which factors predict risk but also how these patterns evolve during the course of a conversation. These findings underscore the importance of explainable, theory-informed tools that can support personalized and context-sensitive approaches to SR assessment.

Consistent with prior research, a history of suicide attempts and hopelessness emerged as the strongest predictors of SR for both men and women (33, 34). However, several psychological risk factors demonstrated gender-specific patterns. Among women, loneliness was a modest but consistent predictor of SR throughout the chat sessions. This finding aligns with prior literature suggesting that women may be more emotionally attuned to relational disconnection, and that perceived social isolation can be a potent driver of suicidal distress in this group (35). In contrast, among men, loneliness was not predictive of SR—a finding that diverges from several large-scale population-based studies, which have shown loneliness to be particularly harmful for men (36, 37).

One possible explanation lies in the contextual and methodological differences: while survey-based studies often measure loneliness via standardized self-report instruments, our findings are drawn from real-time, anonymous help-seeking behavior in digital crisis chats. In such settings, women may be more inclined to articulate feelings of loneliness, while men may be less likely to disclose or even recognize this state during emotionally charged interactions. Instead, men may express interpersonal distress through more cognitive or indirect pathways—reflected in our finding that thwarted belongingness, a perception of being excluded or unneeded, was predictive of SR in men, especially as the conversation progressed. Taken together, these findings underscore the importance of context-sensitive, gender-informed approaches to SR detection.

Interestingly, depression was only significant among men and only at the final stage of the chat, suggesting that emotional states associated with SR may intensify as men open up gradually in anonymous conversations. These gendered patterns emphasize the need for dynamic, process-based assessment models that account for evolving emotional expression. More generally, our temporal analysis revealed that most psychological predictors, including hopelessness and self-harm, showed increased odds ratios over the course of the chat. This pattern likely reflects the progressive nature of disclosure in digital helpline interactions, where users tend to reveal more severe thoughts and emotions later in the session (38). The gender-specific timing of certain predictors—such as the late emergence of depression in men and the early consistency of loneliness in women—suggests that SR unfolds differently within crisis conversations and may require gender-adapted engagement strategies.

Our age-stratified analyses underscore the developmental specificity of SR factors. Previous suicide attempts were strongest among adolescents, consistent with evidence that early attempts confer elevated future risk (39). Hopelessness and self-harm peaked in late adolescence and early adulthood, a period characterized by identity transitions and heightened vulnerability (40). In contrast, thwarted belongingness became more salient in adulthood, while loneliness predicted risk only among adults over 41, aligning with research on isolation in later life (41). These temporal patterns may support real-time decision-making in crisis settings by highlighting which risk markers typically emerge early in a conversation and may warrant earlier supervisory consultation or focused assessment. Interestingly, Bullying/Cyberbullying, and LGBTQ identity related stressors were inversely associated with SR. This finding is best understood within the context of our data source: Since everyone contacting the helpline is already experiencing emotional distress, these factors likely function as indicators of psychosocial distress but are less effective at discriminating the specific outcome of SR itself, especially when controlling for more proximal predictors like Hopelessness. They are highly prevalent reasons for seeking help, causing them to disproportionately “load” onto the Emotional Distress category. This suggests that while these factors are undeniably crucial for intervention and support, their utility as acute triage markers may be limited in distress settings. These findings highlight the necessity of age-sensitive frameworks in suicide prevention, as the weight of risk factors shifts across the age span.

Taken together, our findings highlight how explainable AI can bridge the gap between psychological theory, real-world crisis communication, and clinical screening needs. By revealing distinct temporal and demographic trajectories of risk factors, the study shows how interpretable models can inform gender- and age-sensitive prevention strategies while maintaining transparency for practitioners. Beyond academic insight, this work illustrates the translational potential of explainable NLP pipelines as digital screening aids that can be integrated into healthcare workflows, crisis helplines, and primary care triage systems. By enabling providers to detect escalating risk earlier, understand *why* a model signals risk, and tailor responses accordingly, explainable AI holds promise for improving both the accuracy and the equity of suicide prevention.

## 5 Limitations

This study has several limitations that should be considered when interpreting the findings, but each also highlights opportunities for future work in explainable AI for suicide prevention.

First, the data were drawn from a single national digital helpline, which may limit generalizability to individuals who do not seek help online or who use different crisis platforms. Future research should expand to multi-platform and cross-cultural datasets, allowing explainable AI models to capture broader patterns of SR expression.

Second, demographic characteristics were self-reported and unverified, raising the possibility of misclassification. While such noise is common in large-scale, real-world datasets, future AI approaches could integrate multiple metadata sources (e.g., passive digital traces, longitudinal follow-up) to improve demographic accuracy while maintaining privacy.

Third, the lexicon-based NLP approach, though interpretable, cannot capture the full variability of language. This limitation underscores the need to combine transparent lexicon methods with more flexible but still explainable AI models (e.g., attention-based deep learning with interpretable outputs) to balance nuance with clinical usability.

Fourth, the cross-sectional design of chat sessions precludes longitudinal inference. Each session provides a snapshot of psychological states, but explainable dynamic risk modeling could be developed by integrating follow-up outcomes, enabling personalized, temporally sensitive AI screening tools.

Fifth, the study's age categories reflect the demographic context in which the helpline operates. In this setting, ages 18–20 typically coincide with mandatory military service, shaping the life circumstances and stressors common in this developmental period. Because age-related trajectories vary across countries, the patterns observed in this study may not generalize directly to settings with different developmental or societal structures.

## 6 Conclusions and implications

This study demonstrates the value of integrating psychological theory with explainable AI methods to advance our understanding of how SR is expressed in real time across diverse populations. By analyzing thousands of anonymous crisis chats, we identified key psychological risk factors and their trajectories across gender and age groups, showing that constructs such as hopelessness, self-harm, loneliness, and thwarted belongingness vary in salience depending on demographic context and conversation stage. These findings underscore the importance of personalized, dynamic approaches to suicide prevention that account for how distress is communicated differently by men and women, and across the life course. Beyond clinical insights, this work illustrates the potential of transparent, language-based digital tools to support timely and context-sensitive detection of SR in real-world settings. The gender- and age-specific insights generated here provide an empirical foundation for the development of scalable, real-time AI tools that can monitor psychological risk factors during digital crisis interactions. By enabling counselors to detect escalating risk earlier and tailor their responses, such approaches hold promise for improving the accuracy, timeliness, and equity of suicide prevention in both clinical practice and public health.

## Data Availability

The raw data supporting the conclusions of this article will be made available by the authors, without undue reservation.

## References

[1] World Health Organization. Suicide Worldwide in 2019. Geneva, Switzerland: World Health Organization (2021). Available online at: https://www.who.int/publications/i/item/9789240026643 (Accessed August 22, 2025).

[2] Franklin JC Ribeiro JD Fox KR Bentley KH Kleiman EM Huang X . Risk factors for suicidal thoughts and behaviors: a meta-analysis of 50 years of research. Psychol Bull. (2017) 143:187–232. doi: 10.1037/bul0000084 27841450

[3] Favril L Yu R Uyar A Sharpe M Fazel S. Risk factors for suicide in adults: systematic review and meta-analysis of psychological autopsy studies. Evid Based Ment Health. (2022) 25:148–55. doi: 10.1136/ebmental-2022-300549 36162975 PMC9685708

[4] Tang X Wang R Wu C Zhang Y. Analysis and evaluation of explainable artificial intelligence on suicide risk assessment. Sci Rep. (2024) 14:53426. doi: 10.1038/s41598-024-53426-0 38485985 PMC10940617

[5] Thomas S Elzinga N Spathis D Kim CH Wiggins JL Morrissey R . Using transformer-based machine learning and explainability methods to predict suicidal ideation in youth crisis hotline conversations: model development and evaluation study. JMIR Ment Health. (2025) 12:e65280. doi: 10.2196/63809

[6] Huang G Li Y Jameel S Long Y Papanastasiou G. From explainable to interpretable deep learning for natural language processing in healthcare: how far from reality? Comput Struct Biotechnol J. (2024) 24:362–73. doi: 10.1016/j.csbj.2024.05.004 38800693 PMC11126530

[7] Di Martino F Delmastro F. Explainable AI for clinical and remote health applications: a survey on tabular and time series data. Artif Intell Rev. (2023) 56:5261–315. doi: 10.1007/s10462-022-10304-3 36320613 PMC9607788

[8] Canetto SS Sakinofsky I. The gender paradox in suicide. Suicide Life Threat Behav. (1998) 28:1–23. doi: 10.1111/j.1943-278X.1998.tb00622.x 9560163

[9] Miranda-Mendizabal A Castellví P Parés-Badell O Alayo I Almenara J Alonso I . Gender differences in suicidal behavior in adolescents and young adults: systematic review and meta-analysis of longitudinal studies. Int J Public Health. (2019) 64:265–83. doi: 10.1007/s00038-018-1196-1 30635683 PMC6439147

[10] Mergl R Koburger N Heinrichs K Székely A Tóth MD Coyne J . What are reasons for the large gender differences in the lethality of suicidal acts? An epidemiological analysis in four European countries. PLoS ONE. (2015) 10:e0129062. doi: 10.1371/journal.pone.0129062 26147965 PMC4492725

[11] Schrijvers DL Bollen J Sabbe BG. The gender paradox in suicidal behavior and its impact on the suicidal process. J Affect Disord. (2012) 138:19–26. doi: 10.1016/j.jad.2011.03.050 21529962

[12] Gvion Y Levi-Belz Y. Serious suicide attempts: systematic review of psychological risk factors. Front Psychiatry. (2018) 9:56. doi: 10.3389/fpsyt.2018.00056 29563886 PMC5845877

[13] Carballo JJ Llorente C Kehrmann L Flamarique I Zuddas A Purper-Ouakil D . Psychosocial risk factors for suicidality in children and adolescents. Eur Child Adolesc Psychiatry. (2020) 29:759–76. 30684089 10.1007/s00787-018-01270-9 PMC7305074

[14] Esposito-Smythers C Weismoore JT Zimmermann RP Spirito A. Suicidal behaviors among children and adolescents. In:Nock MK, editor. The Oxford Handbook of Suicide and Self-Injury. Oxford, UK: Oxford University Press (2014). p. 61–81.

[15] Gvion Y Apter A. Evidence-based prevention and treatment of suicidal behavior in children and adolescents. In:O'Connor RC Pirkis J, editors. The International Handbook of Suicide Prevention. 2nd ed. Oxford, UK: Wiley-Blackwell (2016). p. 303–22. doi: 10.1002/9781118903223.ch17

[16] Mościcki EK. Suicidal behaviors among adults. In:Nock MK, editor. The Oxford Handbook of Suicide and Self-Injury. Oxford, UK: Oxford University Press (2014). p. 82–112.

[17] Turecki G Brent DA. Suicide and suicidal behaviour. Lancet. (2016) 387:1227–39. doi: 10.1016/S0140-6736(15)00234-2 26385066 PMC5319859

[18] Qin P Syeda S Canetto SS Arya V Liu B Menon V . Midlife suicide: a systematic review and meta-analysis of socioeconomic, psychiatric and physical health risk factors. J Psychiatr Res. (2022) 154:233–41. doi: 10.1016/j.jpsychires.2022.07.037 35961179

[19] Sadek J Diaz-Piedra B Saleh L MacDonald L. A narrative review: suicide and suicidal behaviour in older adults. Front Psychiatry. (2024) 15:1395462. doi: 10.3389/fpsyt.2024.1395462 38800059 PMC11117711

[20] Woodward A Wyllie C. Helplines, tele-web support services, and suicide prevention. In:O'Connor RC Pirkis J, editors. The International Handbook of Suicide Prevention. 2nd ed. Oxford, UK: Wiley-Blackwell (2016). p. 490–504. doi: 10.1002/9781118903223.ch28

[21] Hoffberg AS Stearns-Yoder KA Brenner LA. The effectiveness of crisis line services: a systematic review. Front Public Health. (2020) 7:399. doi: 10.3389/fpubh.2019.00399 32010655 PMC6978712

[22] Drexler M. Crisis Chat: Providing chat-based emotional support. In:Mishara BL Kerkhof AJFM, editors. Suicide Prevention and New Technologies. London, UK: Palgrave Macmillan (2013). p. 96–110. doi: 10.1057/9781137351692_8

[23] Mokkenstorm JK Eikelenboom M Huisman A Wiebenga J Gilissen R Kerkhof AJFM . Evaluation of the 113Online suicide prevention crisis chat service: outcomes, helper behaviors and comparison to telephone hotlines. Suicide Life Threat Behav. (2017) 47:282–96. doi: 10.1111/sltb.12286 27539122

[24] Yip P Chan WL Cheng Q Chow S Hsu SM Law YW . A 24-hour online youth emotional support: opportunities and challenges. Lancet Reg Health West Pac. (2020) 4:100047. doi: 10.1016/j.lanwpc.2020.100047 34327390 PMC8315660

[25] Castillo-Sánchez G Marques G Dorronzoro E Rivera-Romero O Franco-Martín M. De la Torre-Díez I. Suicide risk assessment using machine learning and social networks: a scoping review. J Med Syst. (2020) 44:205. doi: 10.1007/s10916-020-01669-5 33165729 PMC7649702

[26] Joiner TE Jr. Why People Die By Suicide. Cambridge, MA: Harvard University Press (2005).

[27] Van Orden KA Witte TK Cukrowicz KC Braithwaite SR Selby EA Joiner TE Jr. The interpersonal theory of suicide. Psychol Rev. (2010) 117:575–600. doi: 10.1037/a0018697 20438238 PMC3130348

[28] Galynker I. The Suicidal Crisis: Clinical Guide to the Assessment of Imminent Suicide Risk. Oxford, UK: Oxford University Press (2017). doi: 10.1093/med/9780190260859.001.0001

[29] Posner K Brown GK Stanley B Brent DA Yershova KV Oquendo MA . The Columbia-Suicide Severity Rating Scale: initial validity and internal consistency findings from three multisite studies with adolescents and adults. Am J Psychiatry. (2011) 168:1266–77. doi: 10.1176/appi.ajp.2011.10111704 22193671 PMC3893686

[30] Grimland M Benatov J Yeshayahu H Izmaylov D Segal A Gal K . Predicting suicide risk in real-time crisis hotline chats integrating machine learning with psychological factors: exploring the black box. Suicide Life Threat Behav. (2024) 54:416–24. doi: 10.1111/sltb.13056 38345174

[31] Joyce DW Kormilitzin A Smith KA Cipriani A. Explainable artificial intelligence for mental health through transparency and interpretability for understandability. NPJ Digit Med. (2023) 6:6. doi: 10.1038/s41746-023-00751-9 36653524 PMC9849399

[32] Ali S Abuhmed T El-Sappagh S Muhammad K Alonso-Moral JM Confalonieri R . Explainable Artificial Intelligence (XAI): what we know and what is left to attain Trustworthy Artificial Intelligence. Inf Fusion. (2023) 99:101805. doi: 10.1016/j.inffus.2023.101805

[33] Pemau A Marin-Martin C Diaz-Marsa M. de la Torre-Luque A, Ayad-Ahmed W, Gonzalez-Pinto A, et al. Risk factors for suicide reattempt: a systematic review and meta-analysis. Psychol Med. (2024) 54:1897–904. doi: 10.1017/S0033291724000904 38623694

[34] Freichel R Nock MK O'Shea BA. A network outcome analysis of psychological risk factors driving suicide risk in emergency department patients. Nat Ment Health. (2025) 3:346–53. doi: 10.1038/s44220-025-00389-4

[35] Beutel ME Klein EM Brähler E Reiner I Jünger C Michal M . Loneliness in the general population: prevalence, determinants and relations to mental health. BMC Psychiatry. (2017) 17:97. doi: 10.1186/s12888-017-1262-x 28320380 PMC5359916

[36] Shaw RJ Cullen B Graham N Lyall D Mackay D Okolie C . Living alone, loneliness and lack of emotional support as predictors of suicide and self-harm: a nine-year follow-up of the UK Biobank cohort. J Affect Disord. (2021) 279:316–23. doi: 10.1016/j.jad.2020.10.026 33096330 PMC7758739

[37] Pirkis J Spittal MJ Keogh L Mousaferiadis T Currier D. Masculinity and suicidal thinking. Soc Psychiatry Psychiatr Epidemiol. (2017) 52:319–27. doi: 10.1007/s00127-016-1324-2 28025691

[38] Xu Y Kwok HR Lu W Ng RMK Chan CLW Law YW . Identifying the transition point to suicidal ideation in online text-based counselling: a network-based model. Commun Med. (2022) 2:22. doi: 10.1038/s43856-022-00222-435604802

[39] Probert-Lindström S Berge J Westrin Å Öjehagen A Skogman Pavulans K. Long-term risk factors for suicide in suicide attempters examined at a medical emergency in patient unit: results from a 32-year follow-up study. BMJ Open. (2020) 10:e038794. doi: 10.1136/bmjopen-2020-038794 33130567 PMC7783608

[40] Mortier P Auerbach RP Alonso J Axinn WG Cuijpers P Ebert DD . Suicidal thoughts and behaviors among college students and same-aged peers: results from the World Health Organization World Mental Health Surveys. Soc Psychiatry Psychiatr Epidemiol. (2018) 53:279–88. doi: 10.1007/s00127-018-1481-6 29340781 PMC5896296

[41] Bennardi M Caballero FF Miret M Ayuso-Mateos JL Haro JM Lara E . Longitudinal relationships between positive affect, loneliness, and suicide ideation: age-specific factors in a general population. Suicide Life Threat Behav. (2017) 49:90–103. doi: 10.1111/sltb.12424 29210215

